# Effect of Total Flavonoids of *Rhizoma drynariae* on Tibial Dyschondroplasia by Regulating BMP-2 and Runx2 Expression in Chickens

**DOI:** 10.3389/fphar.2018.01251

**Published:** 2018-11-02

**Authors:** Wangyuan Yao, Hui Zhang, Xiong Jiang, Khalid Mehmood, Mujahid Iqbal, Aoyun Li, Jialu Zhang, Yaping Wang, Muhammad Waqas, Yaoqin Shen, Jiakui Li

**Affiliations:** ^1^College of Veterinary Medicine, Huazhong Agricultural University, Wuhan, China; ^2^Hubei Three Gorges Polytechnic, Yichang, China; ^3^University College of Veterinary and Animal Sciences, The Islamia University of Bahawalpur, Bahawalpur, Pakistan; ^4^Faculty of Veterinary and Animal Sciences, The University of Poonch, Rawalakot, Pakistan; ^5^College of Animals Husbandry and Veterinary Medicine, Tibet Academy of Agricultural and Animal Husbandry University, Linzhi, China

**Keywords:** *Rhizoma drynariae*, tibial dyschondroplasia, BMP-2, Runx2, gene expression

## Abstract

Tibial dyschondroplasia (TD) is an abnormality of the growth cartilage that occurs in chickens and other rapidly growing avian species. This disease not only cause huge economic losses, but also greatly affects animal welfare. The total flavonoids of *Rhizoma drynariae* (TFRD) has been used to cure wide variety of diseases including bone fractures and osteoarthritis and osteoporosis. However, less information is available about the using of TFRD against the TD. The aim of this study was to determine the effect of TFRD on TD by regulating BMP-2 and Runx2 in chickens. A total of 200 birds were randomly divided into control, TD, TD recovery (TDR), and TFRD groups. All the groups were given standard diet with an addition of thiram (50 mg/kg) from days 3 to 7 in TD, TDR, and TFRD groups in order to induce TD in chickens. After the induction of TD, the birds of TFRD group were fed standard diet with the addition of TFRD at 20 mg/kg. Clinical results conveyed that TFRD can improve the growth performance of the TD chickens and recover normal activity, and it is more obvious than TDR. Gene expressions of BMP-2 and Runx2 were down-regulated during the development of the disease and were up-regulated obviously after TFRD treatment. In conclusion, TFRD not only decreased the mortality rate but also increased the growth performance of TD in chickens. In conclusion, TFRD plays important role in improving the growth performance, adjusting the relevant physiological indicators, and regulating BMP-2 and Runx2 in chickens.

## Introduction

Tibial dyschondroplasia (TD) is one of the most common skeletal diseases in fast growing broilers (Shahzad et al., 2014; Nabi et al., 2016). It is characterized by an abnormal plug of non-vascularized and un-mineralized with white opaque cartilage dominating the proximal metaphysis of the tibiotarsus and occasionally the tarsometatarsus (Leach and Nesheim, 1965). The pathogenesis of TD is related to variety, growth rate, daily management, toxins, and feed composition (Li et al., 2008; Rekaya et al., 2013). Because the mechanism underlying of TD development is still unclear, the disease causes lameness, bone fractures, and local infection lead to the enormous economic losses and loss of animal welfare (Farquharson, 2002). Generally, previous studies have shown that the skeletal disease is closely related to the formation and differentiation of osteoblasts and osteoclasts (Ruff et al., 2013).

It is studied that thiram is toxic to chickens (Oruc, 2009) and can induce TD (Tian et al., 2013). Due to the provision of three contaminated feeds to broilers, the incidence of TD in chickens has increased significantly, and the symptoms of chickens are similar to natural occurrence of TD (Rath et al., 2007). Several studies have demonstrated that thiram is a potent TD-inducing agent (Iqbal et al., 2016; Zhang et al., 2018b), but the mechanism by which thiram causes TD in broilers is not known.

Traditional Chinese medicines are widely used for prevention and treatment of all kinds of diseases (Wang et al., 2015). It is famous for excellent treatment, low side effects, and wide range of use safety. Total flavonoids from *Rhizoma drynariae* (TFRD) is an herbal product extracted from the dried root of *Rhizoma drynariae* (Liu et al., 2012). TFRD improves the underlying activity of osteoblast and osteoclast by regulating BMP pathways in bone metabolism and so on (Sun et al., 2016). It is a kind of Chinese medicine (Qianggu capsule), that is produced by Qi-Huang Pharmaceutical Co., Ltd. in China. It has been widely used to treat bone fractures, relief the pain, and as renal tonic (Reddi, 2000).

Bone morphogenetic proteins (BMPs) are a growth factor with osteogenic inductiveness, which belongs to the transforming growth factor-βsuper family. BMPs are effective in the cell proliferation, differentiation, and invasion; expect for the induction of bone formation (Okazaki and Sandell, 2004; Salazar et al., 2016). Recently, several studies have reported that most members of BMPs have the function of definite osteogenesis. Currently, BMP-2 is the most widely studied for osteogenic induction (Xiang et al., 2003; Saito et al., 2004).

Runx2 (core binding factor alpha 1) is the key regulator of bone (Stein et al., 2004; Xiao et al., 2004). Runx2 is considered the dominant gene for osteoblast differentiation (Yang et al., 2003). It promotes bone formation and inhibits bone resorption by regulating the expression of specific extracellular matrix protein genes in osteoblasts and the cell cycle of osteoblasts (Komori, 2010).

As mediated osteogenic pathway among the landmark transcription factors, Runx2 regulated by BMPs. The common BMPs pathway, Smad 1/5/8 and Smad 4 bind to the nucleus, and directly involved in the regulation of gene expression (Derynck and Zhang, 2003; Saito et al., 2004). In fact, Runx2 is a specific marker of the osteogenic phenotype of cells as a downstream factor in BMPs. In the BMP-2-mediated osteogenesis pathway, Runx2 is an important specific transcription factor. In osteoblasts, BMP-2 regulates the formation of osteoporosis by regulating these transcription factors, which in turn regulate downstream functional proteins (Bae et al., 2007; Lee et al., 2009). Previously, it has been reported that TFRD increase the BMP-2 and Runx2 expression in tibial growth plate (GP). Therefore, we designed this study to treat TD broilers by TFRD to further investigate its mechanism of action.

## Materials and Methods

### Experimental Materials

Total flavonoids of *Rhizoma drynariae* (TFRD) were purchased from Beijing Qihuang Pharmaceutical Manufacturing Co., Ltd. One-day-old AA broilers were purchased from Jingzhou Zhengda Animal Husbandry Co., Ltd. Thiram purchased in Hebei Zan Feng Biological Engineering Co., Ltd. Trizol was purchased from Introgen. EDTA was purchased from Amresco analytical grade. Ready-to-use SABC immunohistochemical staining kit purchased from Wuhan Boster Company. Reverse transcription, fluorescence quantitative PCR kits were purchased from Beijing TransGen Biotech. Biochemical test kit was purchased from Nanjing Jiancheng Bioengineering Institute. The ^∧^4% neutral formaldehyde fixing solution for the laboratory self-match.

### Experimental Design

All the experiments related to animal trials were approved and maintained to meet the ethics guidelines of Ethics Committee of Huazhong Agricultural University (HZAU), Wuhan, China. A total of 200 1-day-old arbor acres (AA) chickens were randomly divided into four groups: control group, TD group, TD recovery (TDR) group, and TFRD group. The control group was fed the full-price diet and free drinking water daily, and other groups were fed the full-fledged diet on the 3rd to the 7th day with 50 mg/kg Thiram. Furthermore, we used formulated diet according to Zhang et al. (2018c), which was also tested to eliminate the effect and influence of phytoestrogens in the diets from the effects of TFRD. From the 8th day, total of 50 chickens in the TFRD group were orally fed with 20 mg/kg/day TFRD until the end of the experiment. The entire feeding cycle was 18 days. The daily average body weight and average feed intake of each group were recorded.

### Samples Collection and Handling

During this period, 10 chickens from each group were randomly slaughtered by cervical dislocation on day 7, 10, 14, and 18. The width and length of tibias were measured, while the size of tibial GP was calculated using Vernier caliper for each chicken. Furthermore, the tibia, serum, and liver were also collected from each chicken. Half of the tibial cartilage and liver were placed in 4% paraformaldehyde, and the other half were stored in liquid nitrogen at -80°C. All serum samples were frozen at -80°C freezer for future biochemical detection; samples stored in 4% paraformaldehyde for hematoxylin and eosin staining, and immunohistochemistry (IHC). All other samples were stored in -80°C freezer for RT-qPCR analysis.

### Biochemical Indicators and Antioxidant Parameters Detection

The liver plasma was prepared by the homogenization mechanism from liver, and the total superoxide dismutase (T-SOD) and glutathione peroxidase (GSH-Px) were detected according to kit instructions (Nanjing Jiancheng Bioengineering Institute) dubbed the test solution. The values of alkaline phosphatase (ALP) and alanine aminotransferase (ALT) were determined from blood serum. The blood samples were centrifuged at 3000 × *g* for 20 min to separate the serum. The serum samples were processed by using commercial reagent kit. The absorbance of the liquid was record by semi-automatic biochemical detector and presented in U/mg protein (unit per milligram of protein) wet weight of liver tissue. ALP and ALT activities were presented in U/L (unit per liter) according to previous studies (Mehmood et al., 2018).

### Hematoxylin and Eosin (H&E) Staining and Immunohistochemistry

Tibia cartilage in each group of chicks was decalcified in 15% EDTA-2Na solution. After about half a month, the cleaning of bone samples was done by xylene and embedded in paraffin wax. The tissue mass is placed in melted paraffin for embedding. The embedded wax block cut into thin slices by the slicer, usually 3–5 μ thick, finally dewaxed and dyed as described by Mehmood et al. (2018).

The IHC analysis was performed according to Zhang et al. (2018b), the 3% H_2_O_2_ at room temperature for 5–10 min to eliminate endogenous peroxidase activity. After that washed in PBS and incubated with primary antibody for BMP-2/Runx2, then incubated with the secondary antibody. The slides were observed by microscopy.

### RNA Extraction and RT-qPCR

Differences in gene expression between the groups were verified according to the instructions of Applied Biosystems Real-time PCR Systems. The total RNA of GP chondrocytes was extracted by Trizol method. cDNA synthesis kit (Beijing TransGen Biotech) was used to synthesize first-strand cDNA. The reverse transcription was run according to Nabi et al. (2018).

The target gene (BMP-2 and Runx2) and internal reference gene (GAPDH) primers were synthesized by Yingjun Ltd. (Wuhan, China) as given in Table 1. The PCR reactions were run with the StepOne Plus RT-qPCR System using a SYBR Green RT-PCR kit (TransGen, Beijing, China) in a total reaction volume of 20 μl (Mehmood et al., 2017). Reaction conditions were denaturation at 94°C for 30 s, denaturation at 94°C for 5 s, annealing at 58°C for 15 s, extension at 72°C for 30 s, and amplification for 40 cycles.

**Table 1:** Primers used in this study.

Genes	Accession number	Primer sequence (5′-3′)	Product size (bp)
GAPDH	NM_204305.1	F: GCCCAGAACATCATCCCA	137
		R: CGGCAGGTCAGGTCAACA	
BMP-2	XM_015283435.1	F: TCAGCTCAGGCCGTTGTTAG	185
		R: ACCCCACGTCATTGAAGTCC	
Runx2	AF_445419	F: TAAAGGTGACGGTGGATGG	190
		R: TGTGGATTAAAAGGACTTGGTG	


### Statistics

All experimental data were analyzed by SPSS19.0 software. The experimental data were presented as mean ± standard deviation (mean ± SD), and one-way ANOVA is used for the analysis of group differences. The difference among four groups were considered significant if *P* < 0.05.

## Results

### TFRD Prevented the Mortality in TD Chickens

**FIGURE 1:**
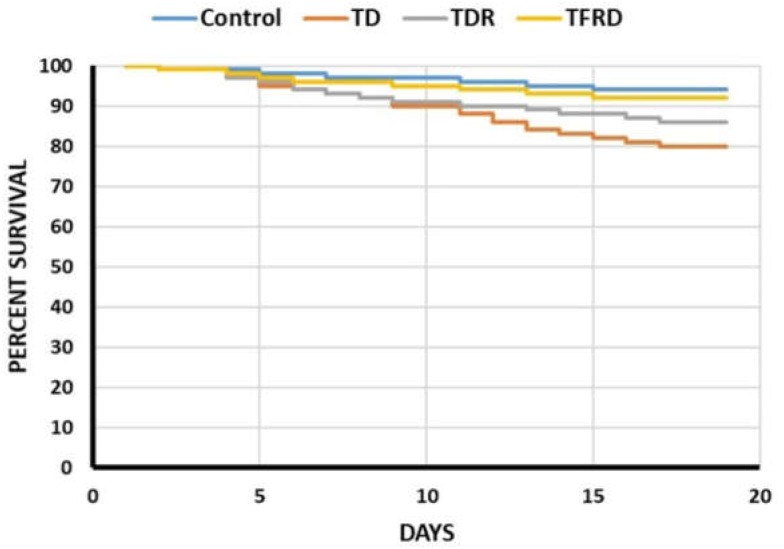
The percentage of survival among control, TD, TDR, and TFRD groups of chickens during the experiment period. The curve was generated by using SPSS22.0.

The mortality was evaluated from days 1 to 18 in four groups. The survival percentage of chickens among TD, TDR, TFRD, and control groups was shown in Figure 1. The results showed that the four polylines have declined in different degrees, but the decreased of TD group was significant as compared with control group and TFRD group. After the administration of TFRD, the tendency of TFRD group was approximately the same as the control group. Although the mortality of TDR group was better than TD group, the results were not significant (Figure 1).

### Chickens Weight Gain

**FIGURE 2:**
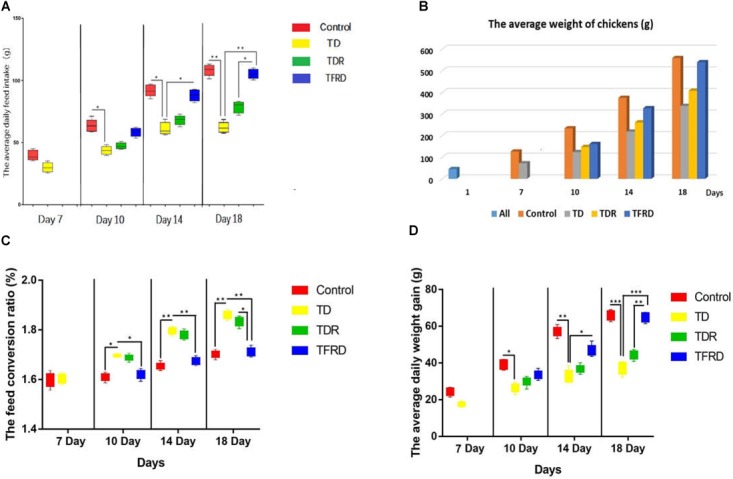
**(A–D)** Average daily feed intake, average weight, feed conversion ratio, and average daily weight gain were recorded on various days 7, 10, 14, and 18 among control, TD, TDR, TFRD groups. All charts are drawn by GraphPad Prism7. The data are expressed as the mean ± SD. ^∗^*P* < 0.05, ^∗∗^*P* < 0.01, ^∗∗∗^*P* < 0.001.

During the period, we detected the average weight of the chickens, the average daily intake, the average daily gain and the feed conversion ratio in each group. The results indicated that the growth performance of TD chickens induced by thiram have significant decreased as compared with the control group. The daily weight, average daily feed intake, and average daily weight gain was significantly reduced in TD group than control group from days 7 to 14. However, the TD broiler chickens gradually recovered and the growth index was closed to the control group after day 14 in TFRD administered group. Although, the growth status of broilers in the TDR group was improved compared with the TD group but the difference was not significant. Meanwhile, there was a significant difference among TDR group, control group, and TFRD group on the day 18 (Figure 2).

### Tibial Bone Parameters Analysis

**FIGURE 3:**
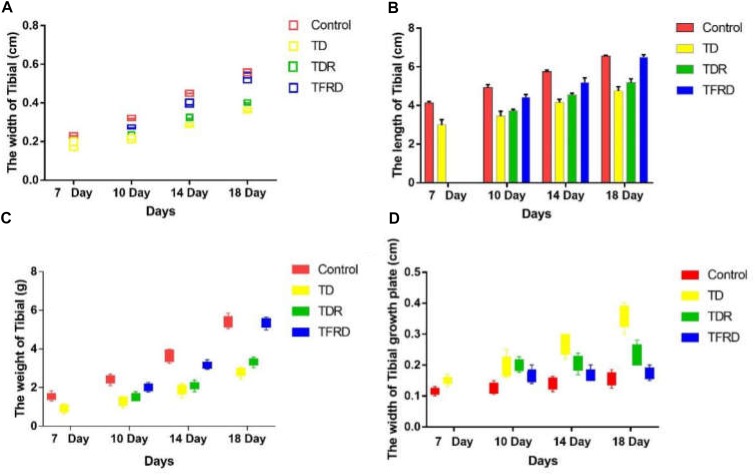
**(A–D)** The overall tibial parameters analysis among control, TD, TDR, and TFRD groups on various days 7, 10, 14, and 18. Width of tibial, length of tibia, weight of tibia, and width of tibial growth plate were recorded with Pearson test by Vernier caliper. The difference was not significant among all the parameters except width of tibial and width of tibial growth plate (*P* < 0.05).

The changes of tibia parameters (length, width, weight, and width of GP) in chickens were measured and recorded among all the groups on days 7, 10, 14, and 18, respectively. As it is shown in Figure 3, all parameters of the control group are better than those of the TD group from days 7 to 18. This strongly proves that TD cause great damage to the tibia of chickens. After administration of TFRD, all the parameters improved gradually, and they were similar to the control group finally. With continuous growth, the chicken has self-healing ability in TDR group especially after day 10. However, the TDR group restored slowly as compared with the TFRD and control group. More importantly, compared to TFRD group, TD and TDR group cannot be completely restored to normal. But, no significant difference between control and TFRD group on day 18.

### Clinical Observation of Chickens

**FIGURE 4:**
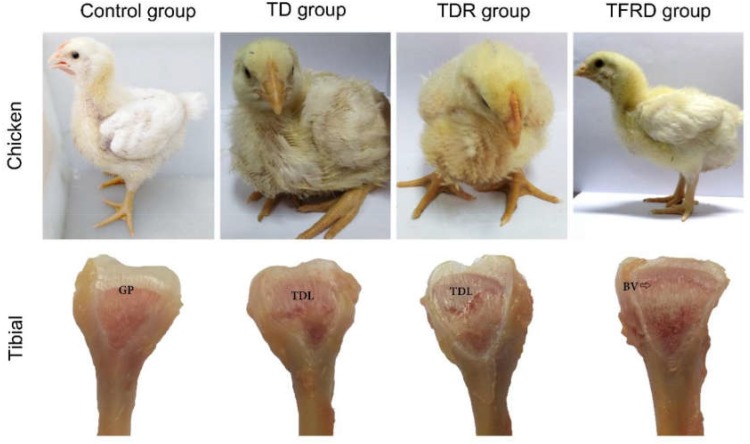
Clinical manifestations and morphological of growth plates in each group. Compared with TD group, the clinical manifestations of control group, and TFRD group were obviously better. Tibial dyschondroplasias of TD and TDR groups show different degrees of damage. The difference was significant between TDR and TFRD groups. BV, blood vessel; GP, growth plate; TDL, tibial dyschondroplasias lesion.

After adding thiram on day 3, broilers started to show signs of lameness. About 90% of the broilers were found with the typical clinical symptoms of TD in thiram-administered groups. This was mainly manifested by difficulty in eating and standing up, not willing to walk or stand in TD group. After day 10, the broilers began to lose their overall health status, the legs were swollen and most of them were unable to stand, even two legs were split in some broilers. However, in TFRD group the broilers started to eat independently and their activity increased after day 8. Until day 18, the broiler’s diet returned to normal. They started to stand completely and can walk freely; they are completely similar to the broilers in the control group. Although broilers have the ability to self-recover, the leg of the broiler still cannot return to normal, the action seems unnatural in TDR group on the day 18. Compared to the control group, the GP of chickens in the TD group was non-vascularized, and there was a white transparent uncalcified embolism from days 7 to 18. The GP gradually returned to normal, with vascular invasion, and reduced calcification embolism in the TFRD-treated group after day 10. The difference was significant between TD and TFRD group on the day 18 (Figure 4).

### Biochemical Indicators Analysis

**FIGURE 5:**
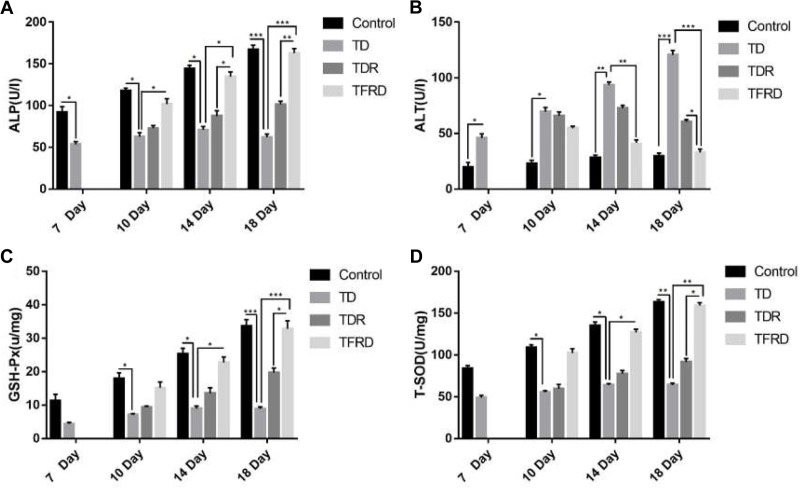
**(A–D)** Biochemical indicator analysis of chickens among control, TD, TD, and TFRD groups on various days 7, 10, 14, and 18. Correlation analysis among T-SOD, GSH-Px, ALP, and ALT by Biochemical test kit. The data are expressed as the mean ± SD. ^∗^*P* < 0.05, ^∗∗^*P* < 0.01, ^∗∗∗^*P* < 0.001.

The serum biochemical indicators of ALP and ALT and liver function indicators of T-SOD and GSH-Px in each group were detected on the days 7, 10, 14, and 18. As shown in Figure 5, the biochemical results of broilers under the influence of TD always were abnormal on day 7 as compared to the control group. Among them, the results of ALP and ALT level were significant different between the TD group and the control group from days 7 to 18. After treatment with TFRD, the biochemical indicators were much better than the TD group on days 10, 14, and 18. At the same time, the broiler chickens in the TDR group also continued to recover, and the corresponding biochemical indicators were also improved, but they remained at a low level until day 18. At the end of the trial, the TFRD group had significant differences compared to the TD and TDR groups. But there was no significant difference between TFRD and control group on day 18.

### Histological Examination of the Tibial Growth Plates

**FIGURE 6:**
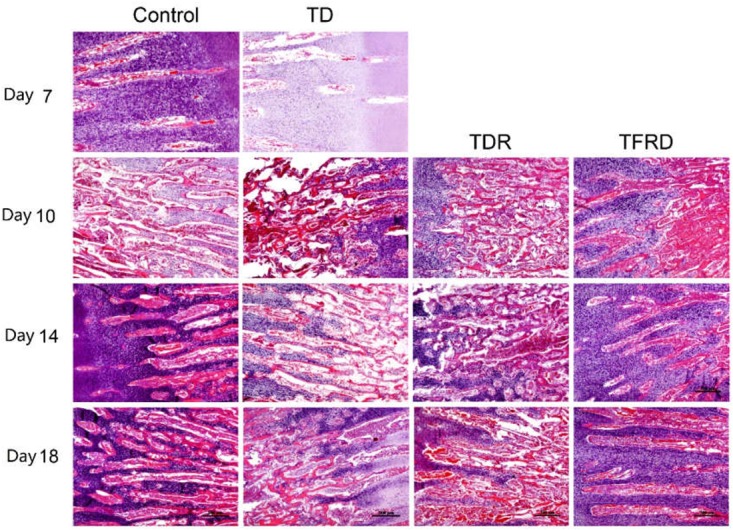
The histopathological micrograph of H&E staining was used to describe the growth and morphological changes of blood vessels in the tibial growth plate on various days 7, 10, 14, and 18. Compared to TD and TDR group, TFRD group showed better results. The difference was not significant between control and TFRD groups.

**FIGURE 7:**
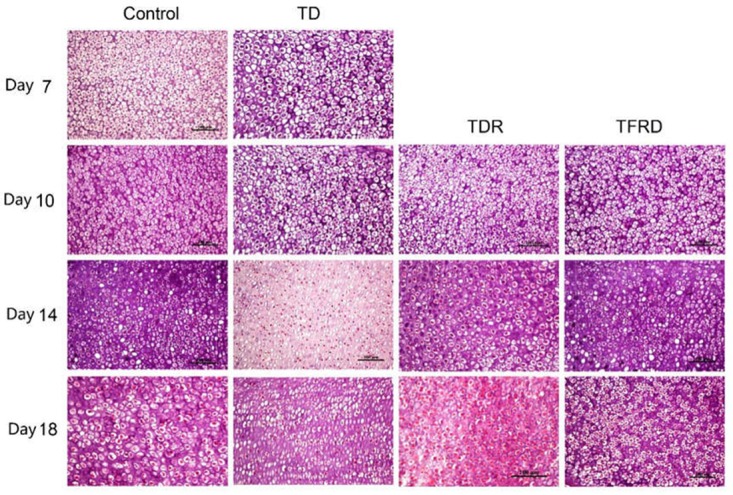
The histopathological micrograph of H&E staining clearly shows the morphology and distribution of cells in GP on days 7, 10, 14, and 18.

Microscopic H&E slides of tibial GP displayed a large number of ordered blood vessels, cartilage cells are arranged neatly, and the cell structure is complete in control group. But in TD group, the blood vessels of the GP were sharply reduced, and the structure was fuzzy. The cartilage cells are arranged in a disorderly manner with incomplete cell morphology, nuclear fragmentation, and nuclear dissolution in TD chickens. After day 7, blood vessels invaded the GP at the beginning and were gradually formed in TFRD treatment group. At the same time, the chondrocytes were repaired and regenerated by TFRD, appeared in a complete, and orderly arrangement in the field of vision. The TDR group presented a slightly better view than the TD group. But neither the morphology of the blood vessels nor the structure of the cells was much in TD group than that in TDR group on the day 18 (Figures 6, 7).

### Immunohistochemistry Analysis of Tibial Growth Plates

**FIGURE 8:**
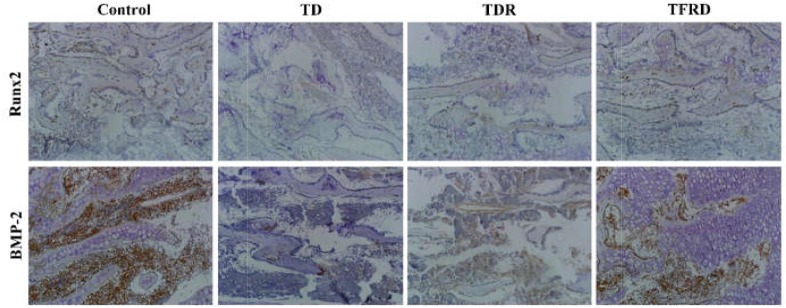
Immunohistochemical localization of BMP-2 and Runx2 in control, thiram, recovery, and TFRD groups. TFRD group has more localization of both BMP-2 and Runx2 than TD and TDR groups. Control group was similar to the TFRD group.

The IHC assay detected the expression of BMP-2 and Runx2 antibodies in tibial GP. Our results clearly showed that both BMP-2 and Runx2 have low expression in the cartilage cells of TD group compared to the control group. While the expression of BMP-2 and Runx2 were increased in TFRD group. No difference in antibody expression between TDR and TD group (Figure 8).

### Expression of BMP-2/Runx2 Genes in Tibial Growth Plates

**FIGURE 9:**
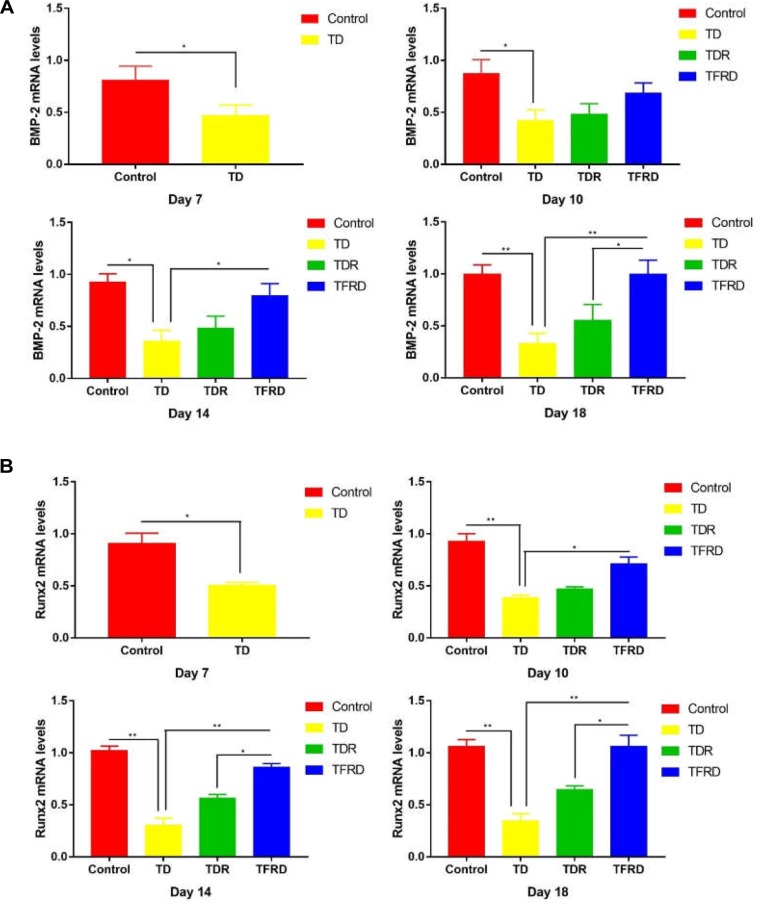
**(A)** The mRNA levels of BMP-2 gene in growth plates (GPs). **(B)** The mRNA levels of Runx2 genes in GPs were detected by Real-time quantitative PCR on days 7, 10, 14, and 18. GAPDH was used as the control gene to display the mRNA levels of the target (BMP-2 and Runx2) gene. The results are expressed in arbitrary units as the means ± SEM. ^∗^*P* < 0.05, ^∗∗^*P* < 0.01.

To determine the expression of related genes in broilers when they were suffered from TD and treated with TFRD, the mRNA expression of genes involved in the GP of chickens were examined on days 7, 10, 14, and 18 in each group. The results revealed that both BMP-2 and Runx2 expression were significantly down regulated from days 7 to 14 in TD-afflicted group as compared to control group. However, TFRD treatment significantly increased the BMP-2 and Runx2 expressions levels in GP cartilage as compared with TD group. It is worth mentioning that the gene expression levels of BMP-2 and Runx2 in TDR group were higher than that of the control group on day 18. The genes expression of BMP-2 and Runx2 in TDR group was significantly lower than that of TFRD group after day 10 (Figures 9A,B).

## Discussion

With the continuous improvement of people’s living standard, the demand of meat products is increasing. Poultry meat and it by-products play an important role in the market with the increasing status of poultry industry (Scanes, 2007). Among the kinds of poultry diseases, skeletal problems are very intractable and have a great impact on economic loss and animal welfare (Cook, 2000). TD is a skeletal disease, which is researched in nutrition, genetic, cell physiology and biochemistry, angiogenesis, invasion mechanism, and other aspects have conducted the in-depth study, but it mechanism is still unclear. TD is characterized by avascular GPs, lack of mineral deposits, and the appearance of white, opaque cartilage (Tian et al., 2013). The clinical symptoms mainly showed that feed intake is decreased at early stages and then tibia joint swelling, difficulty in standing and gait. What’s more, even lead to death in the last period. The pathogenesis of TD is related to many factors, which are vascular factors and fibroblast growth factor, vitamin D. However, many recent researchers have found that the bone modification and remodeling play a pivotal role in TD pathogenesis (Cao et al., 2012; Liang et al., 2012). TFRD promoted bone formation and repair. TFRD can raise the proliferation of osteoblasts while inhibiting the activity of osteoclasts (Guozhi et al., 2014; Wei et al., 2017). In this study, the TFRD administration to the thiram-induced TD broilers significantly reduced the mortality rate and treated TD broilers in a right way. After TFRD was administered, broilers gradually resume free walking; the expression levels of BMP-2 and Runx2 genes in the GPs of broiler chickens were significantly up-regulated as compared to TD group. Our previous study showed that thiram induced TD by effecting chondrocytes interest in TD disease (Zhang et al., 2018a,b). So, we focused on the changes in the chondrocytes of proximal tibial GP of TD chickens. The results of present study demonstrated that the cells were irregular arranged, having no nucleus and even dead cells (Figure 7). The chondrocytes were repaired and appeared in a complete and orderly arrangement after TFRD administration. Furthermore, previous studies have been denoted that the trabecular properties of tibia were decreased in TD group as compared with control group (Zhang et al., 2018a). In our present study, we find the same results and the trabecular properties of tibia were increased after giving TFRD medicine.

Total flavonoids of *Rhizoma drynariae* is an important active traditional Chinese medicine. *Rhizoma drynariae* is the dry foundation of the water dragon orthopedic and perennial sugarcane plants. It is well known that Chinese medicine is mainly derived from natural medicines and processed products. It is characterized by safe, less side effects, and less irritating to the gastrointestinal tract and liver and kidney. Now a day, Chinese medicines are more and more inclined used to prevent and treat diseases in China. TFRD has been used to treat the osteoporosis, fractures, and other-related skeletal diseases. Many previous studies have reported that the TFRD is an effective treatment to skeletal diseases in sheep, rabbits, and rats (He, 2010; Xu et al., 2010).

Previous studies reported that BMP-2 and Runx2 have connection with bone metabolism and concerned as the important regulatory genes for bone formation and differentiation. As an important member of the BMPs family, BMP-2 has a strong osteoinductive activity, in addition to the induction of osteoblast differentiation; it can directly promote the calcification of cells and induce the formation of new bone formation. BMP-2 stimulates the differentiation of mesenchymal stem cells into osteoblasts by autocrine or paracrine and further promotes the differentiation of osteoblasts into osteoblasts (De Caestecker and Meyrick, 2001; Chenard et al., 2012). Runx2 is a key factor necessary for osteogenic differentiation and bone development of mesenchymal stem cells. The expression of Runx2 is a sign of osteoblast differentiation. In BMP-2-mediated osteogenic pathways, Runx2 is an important specific transcription factor. In osteoblasts, BMP-2 regulates these transcription factors to regulate downstream functional proteins and promote bone formation (Matsubara et al., 2008; Nishimura et al., 2012).

## Conclusion

In conclusion, TFRD has therapeutic effect on TD via preventing the incidence of TD in broiler chickens. The present study demonstrates that the TFRD plays an important role in recovering GP lesion, normalized performance, and adjusting the relevant physiological indicators along with regulating BMP-2 and Runx2 in chickens.

This is the first study that TFRD has been applied to the prevention and treatment of TD; it has up-regulated BMP-2/Runx2 expression in thiram-induced TD chickens. This study provides new ideas for the future study to prevent TD in broilers.

## Author Contributions

JL, WY, YS, and HZ provided the research idea. KM, XJ, MI, AL, JZ, and YW contributed reagents, materials, and analysis tools. WY wrote the manuscript. HZ and KM revised the manuscript. All the authors participated in writing and reviewing the manuscript.

## Conflict of Interest Statement

The authors declare that the research was conducted in the absence of any commercial or financial relationships that could be construed as a potential conflict of interest.
